# Alternative Oxidase Mediates Pathogen Resistance in *Paracoccidioides brasiliensis* Infection

**DOI:** 10.1371/journal.pntd.0001353

**Published:** 2011-10-25

**Authors:** Orville Hernández Ruiz, Angel Gonzalez, Agostinho J. Almeida, Diana Tamayo, Ana Maria Garcia, Angela Restrepo, Juan G. McEwen

**Affiliations:** 1 Instituto de Biología, Universidad de Antioquia, Medellín, Colombia; 2 Cellular and Molecular Biology Unit, Corporación para Investigaciones Biológicas (CIB), Medellín, Colombia; 3 Facultad de Ciencias de la Salud, Institución Universitaria Colegio Mayor de Antioquia, Medellín, Colombia; 4 Medical and Experimental Mycology Group, Corporación para Investigaciones Biológicas (CIB), Medellín, Colombia; 5 Escuela de Microbiologia, Universidad de Antioquia, Medellín, Colombia; 6 Facultad de Medicina, Universidad de Antioquia, Medellín, Colombia; University of Tennessee, United States of America

## Abstract

**Background:**

*Paracoccidioides brasiliensis* is a human thermal dimorphic pathogenic fungus. Survival of *P. brasiliensis* inside the host depends on the adaptation of this fungal pathogen to different conditions, namely oxidative stress imposed by immune cells.

**Aims and Methodology:**

In this study, we evaluated the role of alternative oxidase (AOX), an enzyme involved in the intracellular redox balancing, during host-*P. brasiliensis* interaction. We generated a mitotically stable *P. brasiliensis AOX* (*PbAOX*) antisense RNA (aRNA) strain with a 70% reduction in gene expression. We evaluated the relevance of *PbAOX* during interaction of conidia and yeast cells with IFN-γ activated alveolar macrophages and in a mouse model of infection. Additionally, we determined the fungal cell's viability and *PbAOX* in the presence of H_2_O_2_.

**Results:**

Interaction with IFN-γ activated alveolar macrophages induced higher levels of *PbAOX* gene expression in PbWt conidia than PbWt yeast cells. *PbAOX*-aRNA conidia and yeast cells had decreased viability after interaction with macrophages. Moreover, in a mouse model of infection, we showed that absence of wild-type levels of *PbAOX* in *P. brasiliensis* results in a reduced fungal burden in lungs at weeks 8 and 24 post-challenge and an increased survival rate. In the presence of H_2_O_2_, we observed that PbWt yeast cells increased *PbAOX* expression and presented a higher viability in comparison with *PbAOX*-aRNA yeast cells.

**Conclusions:**

These data further support the hypothesis that *PbAOX* is important in the fungal defense against oxidative stress imposed by immune cells and is relevant in the virulence of *P. brasiliensis*.

## Author Summary

Thermally dimorphic pathogenic fungi are responsible for potentially life-threatening diseases of immunocompetent and immunocompromised individuals. These microorganisms grow as conidia-producing mycelia in the environment, which when inhaled by the host convert to the pathogenic yeast form at 37°C. During adaptation and growth, fungi interact with host immune cells and must cope with defense mechanisms such as imposed-oxidative stress (e.g., reactive oxygen species; ROS). Alternative oxidase (AOX) is an enzyme recently implicated in the reduction of ROS production by the mitochondria when triggered by external stimuli, such as temperature and ROS. During this work we have evaluated the relevance of AOX during infection with *Paracoccidioides brasiliensis*, the etiological agent of one of the most prevalent mycoses in Latin America, paracoccidioidomycosis. We show that *PbAOX* gene expression is stimulated after interaction with alveolar macrophages or in the presence of H_2_O_2_ and is essential for survival against fungicidal activity of both the immune cells and the ROS compound. Moreover, decreasing *PbAOX* gene expression in *P. brasiliensis* led to increased survival of infected mice. Altogether, our data supports a relevant role for AOX in the virulence of *P. brasiliensis*.

## Introduction

An essential event during cell growth and host invasion of pathogenic fungi is to avoid the toxic effects of reactive oxygen species (ROS), such as superoxide (O_2_), hydrogen peroxide (H_2_O_2_) and hydroxyl (OH^−^) radicals. ROS can either be produced by the fungal mitochondrial electron transport chain or derive from host immune cells (e.g., macrophages) during host-pathogen interaction [1], [2], [3]. Independently of its origin, these oxidizing agents may ultimately alter the bioenergetic status of the cell and affect essential metabolic pathways and/or its growth within host tissues, representing a toxic stimulus that decreases fungal survival [4], [5].

Human pathogenic fungi such as *Paracoccidioides brasiliensis*, *Histoplasma capsulatum*, *Candida albicans*, among others, possess a defense mechanism against ROS that attempts to modulate the oxidative attack. These mechanisms include some enzymes such as superoxide dismutase which catalyze dismutation of O_2_^−^ producing H_2_O_2_ and O_2_ and catalases involved in decomposition of H_2_O_2_ to H_2_O [6]. In addition, it has been demonstrated that *Aspergillus fumigatus* produces an alternative oxidase (AOX) that contributes to both reduction of ROS generated by mitochondria and regulation of energy production and metabolism [7]. Recently, Magnani *et al* (2007) suggested that AOX is required for the *A. fumigatus* pathogenicity, mainly for the survival of *A. fumigatus* conidia during host infection and reduction of ROS generated by activated macrophages [1].

*P. brasiliensis*, the causal agent of Paracoccidioidomycosis (PCM) [8], is a thermal dimorphic fungus that at environmental temperature grows as a mold producing conidia (the infectious particle). Once inhaled by the host, these fungal cells reach the lung alveoli were they interact with epithelial cells and alveolar macrophages [9]. At 37°C the transition to the parasitic yeast form occurs; however, disease development depends on both the virulence of the fungal strain and host-related factors [10]. Thus, fungal survival inside the host cells depends on the capacity to respond to external factors, ranging from temperature changes to oxidative stress. In fact, as a facultative intracellular pathogen, *P. brasiliensis* can persist within the macrophage phagolysosomes due to the existence of defense mechanisms that allows the fungus to survive under nutritionally poor environments and in the presence of ROS [11], [12], [13]. Also, *P. brasiliensis* has been shown to express a powerful antioxidant defense system in the presence of ROS-mediated oxidative stress [14].

Recently, Maricato and co-workers (2010) showed that under oxidative stress induced by H_2_O_2_ and in the presence of activated macrophages, *P. brasiliensis* yeast increases the expression of the flavoprotein monoxigenase family, an important group of antioxidative enzymes [2]. In addition, the analysis of the mitochondrial function of *P. brasiliensis* yeasts revealed the existence of an alternative respiratory chain (AOX), previously demonstrated to play an important role in the control of ROS and other oxidative molecules [7], [15], [16]. Campos *et al* (2005) described in *P. brasiliensis* several genes in an expressed sequence tag (EST) database encoding proteins involved in antioxidant defense, such as catalase, superoxide dismutase, peroxiredoxin, and cytochrome c peroxidase, among others [17]. More recent studies in *P. brasiliensis* have suggested the role of *AOX* gene in the mycelia to yeast differentiation process and in the maintenance of intracellular redox balancing [18].

In the present study, we have evaluated the role of an AOX in *P. brasiliensis* during host-pathogen interaction. To achieve this we used a *P. brasiliensis AOX-*antisense (*PbAOX*-aRNA) strain to elucidate the role of this enzyme during interaction with alveolar macrophages, considered one of the first lines of defense against this human pathogen [19], [20], as well as in a mouse model of infection.

## Methods

### Microorganisms and culture media

*P. brasiliensis* yeast cells (*P. brasiliensis* ATCC 60855) were maintained at 36°C by sub-culturing in Brain Heart Infusion (BHI) media supplemented with 1% glucose (Becton Dickinson and Company, Sparks, MD, USA). Unless indicated otherwise, yeast and mycelia cells were grown in BHI liquid medium at 36°C and 20°C respectively, with aeration on a mechanical shaker and were routinely collected during their exponential phase of growth (72–96 h) [21]. Morphological transition from yeast-to-mycelia was performed in BHI liquid medium at 20° [21]. *P. brasiliensis* conidia were produced as described by Restrepo *et al* (1986). For the assays, conidia were purified using the glass-wool filtration protocol [22] with cell quantification and viability being determined with Neubauer chamber and ethidium bromide-fluorescence staining procedures, respectively [23]. To evaluate cell morphology, yeast cells were exponentially grown, collected, and fixed in a slide and visualized with an Axiostar Plus (Zeiss) microscope.

*Agrobacterium tumefaciens* strain LBA1100 was used as recipient for the binary vectors constructed in this study [24]. Bacterial cells were maintained at 28°C in Luria Bertani (LB) medium containing kanamycin (100 mg/ml). *Escherichia coli* XL-1-Blue strain was grown at 37°C in LB medium supplemented with appropriate antibiotics and was used as host for plasmid amplification and cloning [25].

### Construction of *P. brasiliensis PbAOX*-aRNA strains

DNA from wild-type *P. brasiliensis* (PbWt) exponentially growing yeast cells was extracted using TRIzol® (Invitrogen, USA). Platinum® *Taq* DNA Polymerase High Fidelity (Invitrogen, USA) was employed to amplify aRNA oligonucleotides for *PbAOX* from PbWt DNA: AS1 (100 bp), AS2 (107 bp), and AS3 (162 bp).

Plasmid construction for aRNA gene repression and *Agrobacterium tumefaciens*-mediated transformation (ATMT) of *P. brasiliensis* were performed as previously described [26], [27]. The amplified *PbAOX*-aRNA oligonucleotides were inserted into the pCR35 plasmid under the control of the calcium-binding protein (CBP-1) promoter region from *H. capsulatum* [28]. The pUR5750 plasmid was used as a parental binary vector to harbor the aRNA cassette within the transfer DNA (T-DNA). The constructed binary vectors were introduced into *A. tumefaciens* LBA1100 ultracompetent cells by electroporation [29] and isolated by kanamycin selection (100 mg/ml).

ATMT of *P. brasiliensis* yeast cells was performed using the *A. tumefaciens* cells harboring the desired binary vector as described by Almeida *et al* (2007). Selection of *P. brasiliensis* transformants was performed in BHI solid media with hygromycin B (Hyg; 50 mg/ml) during a 15-day incubation period at 36°C. Randomly selected Hyg resistant transformants were tested for mitotic stability and decrease in *PbAOX* gene expression was analyzed after consecutive subculturing for 15, 30, 60, and 120 days. *P. brasiliensis* yeast cells were also transformed with the empty parental vector pUR5750 as a control during assays carried out in this study.

### Interaction of *P. brasiliensis* with alveolar macrophages

A cell line (MH-S), which corresponds to mouse alveolar macrophages transformed with SV40, was obtained from the European Collection of Cell Cultures (ECACC No 95090612). IFN-g-activated alveolar macrophages were grown in RPMI 1640 medium+2 mM Glutamine (Invitrogen, Carlsbad, CA, USA)+0.05 mM 2-Mercaptoethanol (Sigma Aldrich, USA)+10% fetal bovine serum (Invitrogen, Carlsbad, CA, USA) For the assays, we used confluent monolayers obtained by adding 4×10^5^ cells per well to 24-well tissue culture plates (Nunc, Kamstrup, Denmark) incubated for 24 h at 36°C with 5% CO_2_ prior to evaluating interaction with PbWt and *PbAOX*-aRNA conidia and yeast cells. IFN-g-activated alveolar macrophages were activated for 18 hours by adding recombinant Mouse IFN-γ (BD Pharmigen. ref 554587) at 100 ng/ml. MH-S cell monolayers (activated and non-activated) were washed once with RPMI 1640 culture medium and co-cultured with *P. brasiliensis* conidia or yeasts at a concentration of 4×10^5^ conidia per well and 8×10^4^ yeast cells per well (corresponding to a ratio of 1∶1 for *P. brasiliensis* conidia∶IFN-γg-activated alveolar macrophages and 1∶5 for *P. brasiliensis* yeasts: IFN-γ-activated alveolar macrophages), and incubated for 15 and 30 min, 1, 3, 6, 12, 24 and 48 h at 36°C, 5% CO_2_. After interaction, IFN-γ-activated alveolar macrophages were lysed by adding H_2_O, then PbWt and *PbAOX*-aRNA cells were removed, and dilutions of these suspensions were plated on BHI plates supplemented with 0.5% glucose, 4% horse serum and EDTA 300 mM and incubated at 18°C as previously described by Kurita *et al.* [30]. These results were compared with the number of conidia or yeast cells added to each well. Percentage of viable cells was expressed as the number of CFUs obtained from each experimental well (*P. brasiliensis* conidia or yeast cells with IFN-γg-activated alveolar macrophages) divided by the number of CFUs in the controls (*P. brasiliensis* conidia or yeast cells alone).

### Gene expression analysis

Total RNA from PbWt and *PbAOX*-aRNA fungal cells was obtained using TRIzol ® (Invitrogen, Carlsbad, CA, USA). Total RNA was treated with DNase I (Invitrogen, Carlsbad, CA, USA) and tested using a conventional PCR with *β-tubulin* primers to confirm the absence of chromosomal DNA contamination [31]; cDNA was synthesized using 2 µg of total RNA with SuperScript III reverse transcriptase according to the manufacturer's instructions (Invitrogen, Carlsbad, CA, USA).

Real-time PCR was done using SuperScript™ III Platinum® Two-Step qRT-PCR Kit with SYBR® Green, according to the manufacturer's instructions (Invitrogen, Carlsbad, CA, USA). The CFX96 Real-Time PCR Detection System (Bio-Rad, Headquarters Hercules, California, USA) was employed to measure gene expression levels. *PbAOX* gene expression was evaluated in both PbWt and *PbAOX*-aRNA conidia and yeast cells alone and after interaction with MH-S cell line at the different time points described above. Melting curve analysis was performed after the amplification phase of real time PCR assays to eliminate the possibility of non-specific amplification or primer-dimer formation. Fold changes in mRNA expression were calculated using the difference between the target gene and b-tubulin (house-keeping gene) using delta-delta-Ct [32]. Each experiment was done in triplicate and the expression level measured by triplicate. The following primers were used: PbAOX, L:agggctgggaaatattctttg and R:cttgggagcaagaggtgct); and *b-*tubulin, L:gtggaccaggtgatcgatgt and R:accctggaggcagtcaca).

To determine cytokine expression we used the same samples and RT-PCR procedures as described above, but in this case we used *ubiquitin* as the housekeeping gene. We evaluated the expression of interleukin (IL)-6, IL-10, IL-12p40 and tumor necrosis factor alpha (TNFα) at different time points (15 and 30 min and 1, 3, 6, 12, 24 and 48 h) of infection; as a control we used non-infected IFN-γg-activated alveolar macrophages. Each experiment was done thrice and the expression level was measured in triplicate. The following primers were used: IL-6, L:acacatgttctctgggaaatcgt and R:aagtgcatcatcgttgttcataca; IL-10, L:tttcaattccctgggggagaa and R:gctccactgccttgctcttatt; IL-12p40, L:caaattactccggacggttca and R:agagacgccattccacatgtc; TNFalpha, L:gccaccacgctcttctgtct and R:tgagggtctgggccatagaac; ubiquitin L:tggctattaattattcggtctgcat and R:gcaagtggctagagtgcagagtaa.

### Mouse model of infection

Isogenic 8-week-old BALB/c male mice, obtained from the breeding colony of the Corporación para Investigaciones Biológicas (CIB), Medellín, Colombia, were used in all experiments and were kept with food and water *ad libitum* [33]. All animals were handled according to the national (Law 84 of 1989, Res No. 8430 of 1993) and international (Council of European Communities and Canadian Council of Animal Care, 1998) guidelines for animal research and the experimental protocols were approved by Corporación para Investigaciones Biológicas (CIB) research ethics committee.

Animals were infected intranasally (i.n.) with 4×10^6^ *P. brasiliensis* conidia cells suspended in PBS buffer from either PbWt or *PbAOX*-aRNA strains; conidia cell viability was always above 95%. Prior to infection, fungal cells were washed thrice with PBS and submitted to Neubauer counting procedures. Separate groups of ten mice were used to compare the percentage of survival over a 265-day period post-challenge.

Groups of eight mice were challenged with PbWt and *PbAOX*-aRNA conidia and evaluated for fungal burden at 3 days and 8 and 24 weeks post-challenge. Groups of mice per time point were examined for colony-forming units (CFU) in lung homogenates. The homogenates were plated on Mycosel supplemented with 0.5% glucose, sealed, and incubated at 18°C; colonies were counted daily until no increase in counts was observed. The CFU counts/g of lung tissue is expressed on a log scale.

### H_2_O_2_-induced oxidative stress

The effect of H_2_O_2_ exogenous treatment on *AOX* gene expression and viability of PbWt and *PbAOX*-aRNA yeast cells were evaluated using 3×10^6^ cells in a final volume of 2 ml of PBS. Yeast cells were treated with different concentrations of H_2_O_2_ (0.015, 0,03, 0,0625, 0,125, 0.25, 0.5, 1.0, and 4.1 M) for 60 min at 36°C with aeration on a mechanical shaker. The reaction was stopped by dilution in 8 ml of cold distilled water. The cells were then centrifuged at 900 g for 10 min and the pellet was resuspended in 0.2 ml of PBS to determine cell viability [34].

### Statistic analysis

Data are expressed as average ± standard error of the mean (SEM), and all assays were done at least three times. All statistical analysis, including analysis of variance (ANOVA), was performed using the SPSS 17.0 statistics program. A *p* value<0.05 was considered statistically significant. Survival rate from two independent infections in a mouse model (n = 10 mice for each *P. brasiliensis* strain) was analyzed using Kaplan Meyer and log rank test.

## Results

### Generation of a *P. brasiliensis PbAOX*-aRNA strain

**Figure 1:**
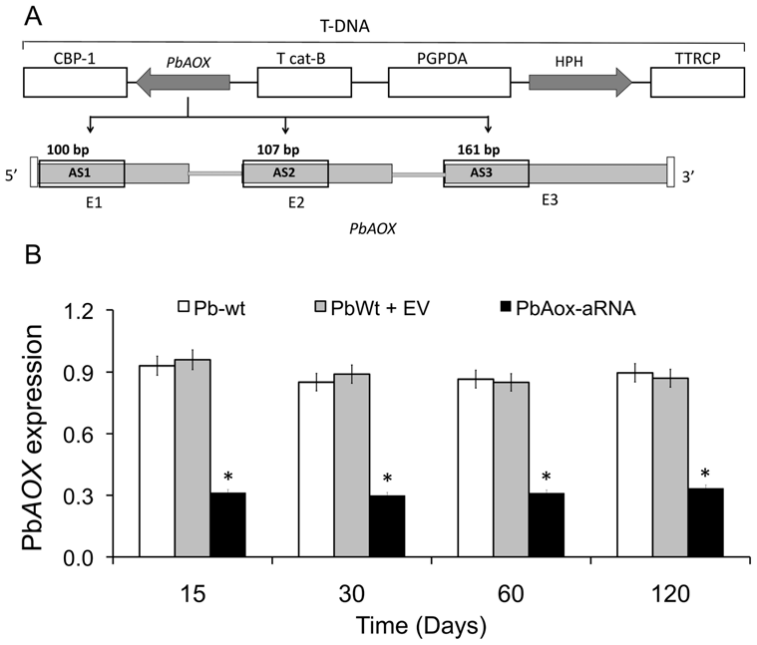
Generation of a *P. brasiliensis PbAOX-*aRNA strain. (A) T-DNA construct for aRNA silencing of *PbAOX* in *P. brasiliensis* via ATMT. *PbAOX-*aRNA oligonucleotides AS1 (base pairs 1 to 100 of *PbAOX*; exon 1), AS2 (base pairs 153 to 260 of *PbAOX*; exon 2), and AS3 (base pairs 99 to 260 of *PbAOX*; exon 3) were amplified, individually placed under the control of the calcium-binding protein (*CBP1*) promoter, and later on inserted into the T-DNA of the binary vector pUR5750 for ATMT of *P. brasiliensis*. (B) Gene expression levels of *PbAOX* in PbWt, PbWt transformed with the empty vector (PbWt+EV), and *PbAOX*-aRNA yeast cells after subculture for 15, 30, 60 and 120 days (gene expression levels obtained by RT-PCR were normalized with the internal reference, TUB2; *: *p*<0.05 when compared with PbWt and PbWt+EV).

A *P. brasiliensis PbAOX*-aRNA strain was generated via ATMT by expressing an aRNA oligonucleotide targeting the coding sequence of the *PbAOX* gene in PbWt yeast cells (Fig. 1A). For control experiments, PbWt yeast cells were independently transformed with the empty vector (PbWt+EV). After confirming mitotic stability, 3 random *PbAOX*-aRNA and empty-vector transformants were selected for gene expression analysis of *PbAOX*. A 70% decrease was detected in all tested *PbAOX*-aRNA strains after 15, 30, 60, and 120 days of sub-culturing without selection of yeast cells, confirming knock-down of gene expression and stable genomic integration of the T-DNA (Fig. 1B). For the functional assays we used the *PbAOX*-aRNA transformant growing after 60 days of sub-culturing (resulting from insertion of the antisense construct AS2).

### *PbAOX* is up-regulated during infection of alveolar macrophages

**Figure 2:**
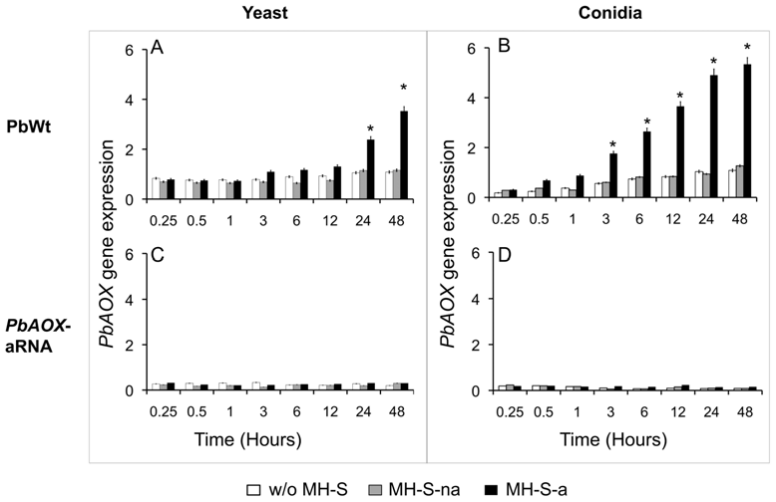
*PbAOX* expression during infection of alveolar macrophages is essential to maintain fungal viability. *PbAOX* expression of cells growing in culture medium (w/o MH-S) and in the presence of non-activated (MH-S-na) or IFN-g-activated (MH-S-a) alveolar macrophages: A. PbWt yeast cells; B. PbWt conidia; C. *PbAOX*-aRNA yeast cells; D. *PbAOX*-aRNA conidia. * P<0.05 when compared with non-activated macrophages.

*PbAOX* expression was evaluated during infection of alveolar macrophages. To serve as a control for subsequent assays, we evaluated *PbAOX* gene expression of yeast and conidia of either PbWt or *PbAOX*-aRNA strains placed alone in macrophage culture medium (Fig. 2). No major alterations were detected in yeast cells of either strain; however, *PbAOX* expression in PbWt conidia cells increased slightly most likely due to the metabolic activation of conidia after being placed in the medium.

No increase in *PbAOX* gene expression was detected after interaction with non-activated IFN-g-activated alveolar macrophages. However, a continuous increase in *PbAOX* gene expression was observed over time in PbWt (yeast and conidia) during interaction with IFN-g-activated alveolar macrophages (Fig. 2A and 2B). *PbAOX* expression in *PbAOX*-aRNA yeast and conidia cells was kept at very low levels throughout all the assays (Fig. 2C and 2D).

### *PbAOX* expression during infection of alveolar macrophages is essential to maintain fungal viability

**Figure 3:**
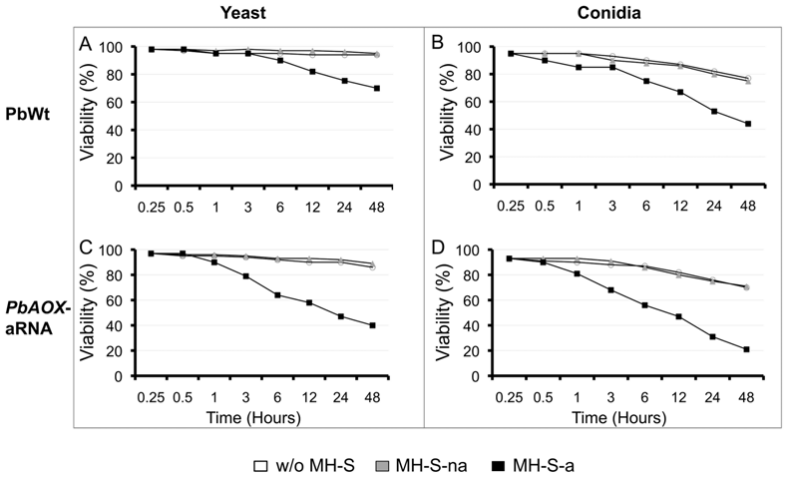
*PbAOX* expression during infection of alveolar macrophages is essential to maintain fungal viability. Fungal viability of cells growing in culture medium (w/o MH-S) and in the presence of non-activated (MH-S-na) or IFN-g-activated (MH-S-a) alveolar macrophages: A. PbWt yeast cells; B. PbWt conidia; C. *PbAOX*-aRNA yeast cells; D. *PbAOX*-aRNA conidia. * P<0.05 when compared with non-activated macrophages.

To elucidate the role of *PbAOX* during oxidative stress response induced by host alveolar macrophages, we determined cell viability upon infection of alveolar macrophages with yeast and conidia cells of the PbWt and *PbAOX*-aRNA strains (Fig. 3). Fungal viability of either PbWt or *PbAOX*-aRNA in the absence of alveolar macrophages and after interaction with non-activated alveolar macrophages was unaltered during all evaluated periods. After 3 h of interaction with IFN-g-activated alveolar macrophages, we observed a decrease in cell viability of yeast and conidia of both strains. Nonetheless, *PbAOX-*aRNA yeast and conidia cells presented a significant decrease in viability when compared to the PbWt strain (Fig. 3D and 3C).

### *PbAOX* does not affect cytokine expression in IFN-g-activated alveolar macrophages

**Figure 4:**
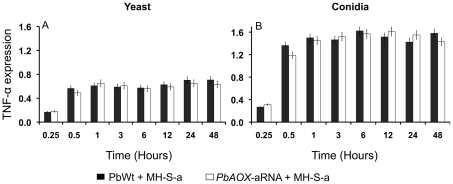
*PbAOX* does not affect cytokine expression in IFN-g-activated alveolar macrophages. TNF-alpha gene expression levels in IFN-g-activated alveolar macrophages (MH-S-a) after interaction with *P. brasiliensis* cells for 0.25, 0.5, 1, 3, 6, 12, 24 and 48 h. A. Interaction with PbWt and *PbAOX*-aRNA yeast cells. B. Interaction with PbWt and *PbAOX*-aRNA conidia. * P<0.05 when TNF alpha gene expression by IFN-g-activated alveolar macrophages is compared after interaction of conidia and yeast cells.

In order to evaluate whether *PbAOX* suppression could alter the cytokine profile in alveolar macrophages, we determined the gene expression of IL6, IL10, IL12p40, and TNF-α during infection of non-activated and IFN-g-activated alveolar macrophages. We did not observe expression for any of the tested cytokines in non-infected alveolar macrophages, either non-activated or activated with IFN-g (data no shown). However, PbWt and *PbAOX*-aRNA yeast and conidia cells were able to induce TNF-α gene expression in IFN-g-activated alveolar macrophages after 30 min of initial interaction (Fig. 4). Interestingly, higher levels of TNF-α were observed during either *PbWt* or *PbAOX*-aRNA conidia-alveolar macrophages interaction when compared to yeast cells after 30 min of infection of IFN-g-activated alveolar macrophages. Expression of IL6, IL10, and IL12p40 were below detection levels for these experiments.

### Decrease in *PbAOX* expression decreases fungal burden in lungs of infected mice and increases survival rate

**Figure 5:**
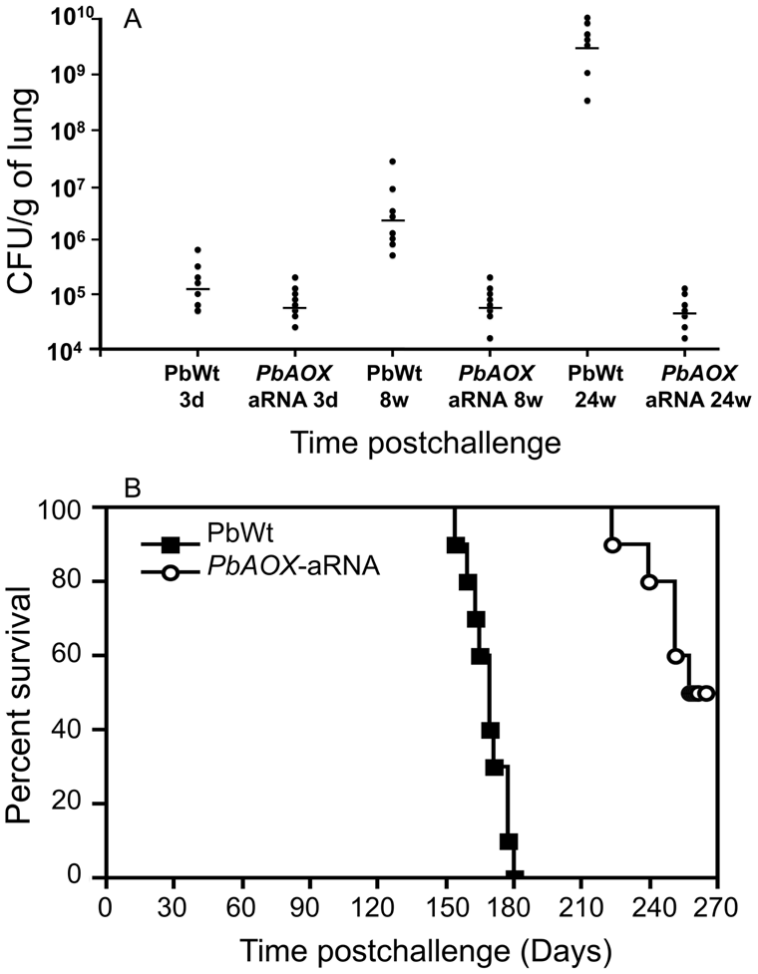
Silencing of *PbAOX* decreases virulence of *P. brasiliensis* in a murine model of infection. A. *P. brasiliensis* colony-forming units (CFU) recovered from pulmonary tissues (n = 8 mice per periods). CFU are expressed as log_10_ per g. of lung. H: hours; w: weeks. * P<0.05 when compared with 3 days after infection. B. Silencing of *PbAOX* decreases virulence of *P. brasiliensis* in a murine model of infection. Representative survival plot of an experimental infection carried out in BALB/c mice (n = 10 mice) challenged i.n. with 4×10^6^ PbWt or *PbAOX*-aRNA conidia cells (*P*<0.0001 when compared with mice infected with PbWt conidia).

The relevance of *PbAOX* during host-pathogen interaction was further evaluated using a mouse model of infection challenged with conidia. We did not observe a significant difference in the colony-forming units (CFU) counts recovered from lungs of mice three days after infection with PbWt or *PbAOX*-aRNA conidia (Fig. 5A). However, 8 and 24 weeks post-challenge, lower CFU counts in lungs of mice infected with *PbAOX*-aRNA conidia were significant lower and similar to those recovered at 3 days post-challenge. Contrarily, fungal burden was significantly higher in the lungs of mice infected with PbWt conidia at the different evaluated periods (Fig. 5A).

Regarding survival, animals infected with the PbWt strain started to die at day 154, with an average survival of 167 days, while mice infected with the *PbAOX*-aRNA strain showed an increased survival time starting to die at day 223 with a survival average of 267 days (*P*<0.0001; Fig. 5B).

### *PbAOX* is involved in the detoxification process exerted by exogenous H_2_O_2_

**Figure 6:**
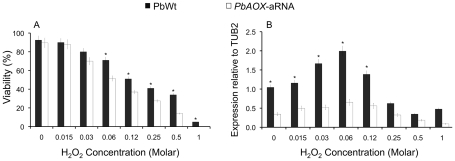
*PbAOX* plays an important role in the detoxification of H_2_O_2_. (A) Cell viability of PbWt and *PbAOX-aRNA* after incubation for 1 hour at different concentrations of H_2_O_2_. (B) *PbAOX* gene expression after incubation for 1 hour at different concentrations of H_2_O_2_ (gene expression levels obtained by RT-PCR were normalized with the internal reference, TUB2; * indicates *P*<0.05 when compared with *PbAOX-aRNA* strains).

Given the role AOXs can play to dampen ROS production by mitochondria [35], we evaluated the viability of *P. brasiliensis* yeast cells from PbWt and *PbAOX*-aRNA strains when exposed to different concentrations of H_2_O_2_. Although systematic differences were consistently registered at all concentrations of H_2_O_2_, they were significant only for concentrations higher than 0.0625 M (Fig. 6A). Importantly, PbWt yeasts cells highly expressed *PbAOX* when exposed to exogenous oxidative stress, peaking at 0.0625 M of H_2_O_2_, but decreasing at higher concentrations (Fig. 6B). Only a minimal but not significant increase in the *PbAOX* expression levels was detectable in *PbAOX*-aRNA yeast cells upon exposure to H_2_O_2_ (at 0.0625 M).

## Discussion

It is well documented that ROS (e.g., O_2_^−^, H_2_O_2_ OH^−^) are produced as a part of host defense mechanisms against fungal pathogens, including *P. brasiliensis* [18], [36], and that these molecules induce damage of mitochondrial DNA [37]. Pathogenic fungi possess mechanisms to attenuate the cell damage induced by ROS observed during host adaptation and growth through the production of detoxifying molecules [18], [38]. Among these is alternative oxidase (AOX), an enzyme that is part of a non-protonmotive non-energy-conserving pathway of the mitochondrial respiratory process and plays a role in lowering ROS formation in fungi and plant mitochondria [39], [40]. AOX couples oxidation of ubiquinone to the reduction of O_2_ to H_2_O and its activation is thought to be triggered by diverse external stimuli, such as temperature and ROS [4], [14], [15], [40]. In *P. brasiliensis*, AOX has been associated not only to the reduction of mitochondrial-generated ROS and maintenance of intracellular redox balance in yeast cells but also a putative role during the mycelia to yeast morphological switch [18]. In the present study, we have explored the involvement of *PbAOX* during interaction of *P. brasiliensis* yeast and conidia with host alveolar macrophages and in the presence of exogenous H_2_O_2_, as well as in a mouse model of infection. To support our data, we generated a *P. brasiliensis* strain with consistently reduced *PbAOX* gene expression using aRNA technology [26], [27], [41] to compare with the PbWt strain (Fig. 1).

INF-g-activated alveolar macrophages are able to produce ROS that are important effectors against infecting fungi [11], [20], [42]. *In vitro* assays revealed that the decrease in *PbAOX* expression led to increased susceptibility against the fungicidal activity of INF-g-activated alveolar macrophages (Fig. 3). This susceptibility was even greater in conidia than in yeast cells revealing a differential response depending on the fungal morphotype. In addition, a reduction of *PbAOX* mRNA levels significantly decreased survival of *P. brasiliensis* yeast cells exposed to exogenous H_2_O_2_, further supporting a role of this enzyme against oxidative agents (Fig. 6). These results further implicate the *PbAOX* gene in the resistance of *P. brasiliensis* to the oxidative burst generated by alveolar macrophages and are in agreement with previous reports indicating that AOX is required for *A. fumigatus* pathogenicity [1]. As *P. brasiliensis* is an intracellular facultative pathogen, it will be important to understand if PbAoxp is also important as a defense mechanism against host-imposed oxidative stress or for persistence within the phagolysosome.

Interestingly, *PbAOX* gene expression was higher in PbWt conidia than in yeast cells and was only induced in the presence of INF-g-activated alveolar macrophages (Fig. 3). The fact that conidia had to cope not only with INF-g-activated alveolar macrophages but also with the temperature-induced morphological switch might explain the diminished survival. Taking in to account that conidia basal metabolic activity is much lower than yeast cells we hypothesize that exposure to 37°C and metabolic activation of the conidia-to-yeast (C-Y) morphological transition generates a higher quantity of ROS as by-products. However, conidia are not yet equipped with the enzymatic machinery to detoxify these molecules as yeast cells possibly explaining the difference in viability (either in the presence or absence of alveolar macrophages). In fact, recent work in our laboratory has shown that *PbAOX* plays an important role in the maintenance of cellular homeostasis during the C-Y transition (manuscript under preparation). Martins and colleagues (2010) have also demonstrated that *PbAOX* plays an important role during the morphological transition [18].

TNF-alpha plays an important role in modulation of immune response against *P. brasiliensis* [20], [43]. Previous reports have shown that this pro-inflammatory cytokine activates macrophages, which exert a fungicidal mechanism independent of nitric oxide production [43], [44]. In the present study, we observed that INF-g-activated alveolar macrophages infected with either fungal morphotype induced expression of TNF-alpha (Fig. 4). Although this cytokine could be implicated in the fungicidal activity of INF-g-activated alveolar macrophages against *P. brasiliensis* through ROS production, we show that reduction of *PbAOX* expression did not affect the production of this cytokine. Interestingly, *P. brasiliensis* conidia induced a higher production of TNF-alpha. This difference could be attributed to the differential cell wall composition of conidia (mainly 1,3-beta glucans) and yeast (mainly 1,3-alpha glucans) cells. It has been demonstrated that 1,3-beta glucans are recognized by the dectin-1 receptor present in macrophages triggering cell activation, including cytokine production such as TNF-alpha [45], [46]. Additionally, our data show that the reduction in expression of *PbAOX* leads to a significantly increased survival rate in a mouse model of infection (Fig. 5). Also, fungal burden in mice challenged with *PbAOX*-aRNA conidia were lower at all the evaluated periods, suggesting that *PbAOX* is important for the establishment of the infection, namely during the initial phase when fungal cells must face the temperature-induced morphological shift to the yeast form while simultaneously interacting with immune cells (e.g. alveolar macrophages) that are able to induce an oxidative stress response (e.g. ROS). The relevance of AOX during infection was previously reported in other human pathogenic fungi such as *Cryptococcus neoformans* and *Aspergillus fumigatus* [1], [47]. This further suggest that this enzyme may play an important role in the adaptation of *P. brasiliensis* and development of the disease, possibly by improving survival within phagocytic cells [1], [6], [47].

Altogether, evidence presented throughout this work support the relevance of *PbAOX* in the virulence of *P. brasiliensis*. Taking into consideration that AOX has also been shown to cause decreased susceptibility of *Candida albicans* to the antifungal drug fluconazole [3], *PbAOX* may represent a putative target for treatment of paracoccidioidomycosis. Work in our laboratory is being finalized to further contribute to the elucidation of biochemical events associated to AOX's role in *P. brasiliensis* life cycle.
